# Cold Atmospheric Pressure Plasma Comb—A Physical Approach for Pediculosis Treatment

**DOI:** 10.3390/ijerph16010019

**Published:** 2018-12-21

**Authors:** Lars ten Bosch, Birgit Habedank, Dominik Siebert, Julia Mrotzek, Wolfgang Viöl

**Affiliations:** 1 University of Applied Sciences and Arts HAWK, Faculty N, Von-Ossietzky-Strasse 99/100, 37085 Göttingen, Germany; dominik.siebert@hawk.de (D.S.); julia.mrotzek@hawk.de (J.M.); wolfgang.vioel@hawk.de (W.V.); 2 German Environment Agency, Corrensplatz 1, 14195 Berlin, Germany; birgit.habedank@uba.de

**Keywords:** CAPP, *Pediculus humanus*, head lice, body lice, pediculosis, physical treatment, plasma-based pest management, plasma comb

## Abstract

Pediculosis, that is the infestation of humans with *Pediculus humanus capitis* (head lice), poses a worldwide problem that is as old as mankind itself. Over the centuries, man has developed a variety of remedies, all of which have ultimately culminated in the use of chemical agents. Some of these remedies are known to produce successful results. A large portion of the effective remedies used to kill lice and their eggs contain insecticides, but there is an increasing number of reports of head lice populations revealing an increased resistance. This study presents an alternative treatment approach, the efficacy of which is based on physical effects. Cold atmospheric pressure plasmas have successfully shown their formidably wide application range within the field of plasma medicine. This study presents a plasma device in its current stage of development that is engineered as a consumer product to enable an alternative physical and insecticide-free option for the treatment of pediculosis. An efficacy study concerning different developmental stages of *P. humanus humanus* is presented. *P. humanus humanus* was chosen as a substitute test organism for *P. humanus capitis* due to possible laboratory rearing and high anatomic similarity. The study shows how a single stroke of the plasma device over a hair strand (approximately 22 cm in length with a weight of 1.5 g) led to mortality rates of 68.3% (50.0; 79.7) (95% CI) in the juvenile test group, a mortality rate of approx. 67.7% (54.9; 78.8) (95% CI) in the female test group, and approx. 46.7% (28.3; 65.7) (95% CI) in the male test group. When single eggs were introduced directly into the plasma for approx. 1 s, younger eggs (0–2 d) showed a higher mortality of 66.7% (42.7; 82.7) than the older (4–6 d) eggs, with 16.7% (5.6; 34.7) (CI). Furthermore, the results of a risk assessment of the device are described. The article concludes with necessary handling instructions as well as further developmental steps, derived from the results of the efficacy and the risk assessment study.

## 1. Introduction

Over the course of the last few decades, increasing numbers of human lice globally have developed resistance to the active substances of various different classes of insecticides. Although there are currently many different products on the market, ranging from pediculicides with pharmacological mode of action to various medical devices to treat head lice, the number of infestations remains high. On the one hand, there is a need to improve the application procedure and user instructions of given remedies [1,2]. On the other hand, the development of new effective methods with alternative modes of action is needed to safely eradicate lice infestations. Products developed for the control of lice on humans must meet three main requirements. Firstly, the applied pediculicide or procedure must have a sufficiently high toxicity or killing effect to eradicate the lice population, while secondly, the toxicity or other adverse effects to the host must be kept at a minimum level. Thirdly, there should be no negative impact to the environment, as is already required for medicinal products, biocides, or pesticides [3]. The second requirement is of utmost importance, as head lice are widespread among children. When treating pediculosis, a high effectivity of the applied treatment at a preferably low impact on the host organism is tantamount, leaving no side effects.

The most important reason for the development of an easy-to-use plasma device for the treatment of pediculosis is the possibility of omission of pesticides. Different clinical and parasitological studies show how quickly lice can adopt to the use of different agents. Durand et al. [4] showed how, due the alteration of binding sites within the treated lice as well as strongly reduced knockdown resistance, conventional topical pediculicides (neurotoxic insecticides) have suffered considerable effectivity loss globally [4,5]. Durand et al. concluded, in particular, that resistance to synthetic pyrethroids has become prominent due to their extensive use. With the present study, we hope to give some first impressions of atmospheric pressure plasmas as a possible treatment alternative to the conventional agents.

To overcome the threat of resistance development in lice, a physical treatment method was developed based on the concept of cold atmospheric pressure plasma (CAPP). As a key technology, CAPPs are currently used for purposes of surface modifications, like enhancement of wettability [6,7,8], precision cleaning purposes [9,10,11], or within the field of plasma medicine [12,13,14]. Known to be capable of interacting with biological surfaces and wounds, CAPPs have been developed to treat different diseases, ranging from skin diseases (e.g., [15,16,17,18]) to different cancer treatment approaches (e.g., [19,20,21]). Here, we present first results on CAPP as a prominent, pain-free tool for the treatment of pediculosis. The basic effectivity of a CAPP application using a handheld device to kill *Pediculus humanus* and their eggs is demonstrated. Furthermore, a risk assessment of the device concerning the exposure of possible applicants (i.e., emerging reactive species, possible patient leakage currents (PLCs), and UV radiation) has been measured and assessed. For easier readability, the device is hereinafter referred to as plasma comb (PlaCo).

## 2. Materials and Methods

**Figure 1:**
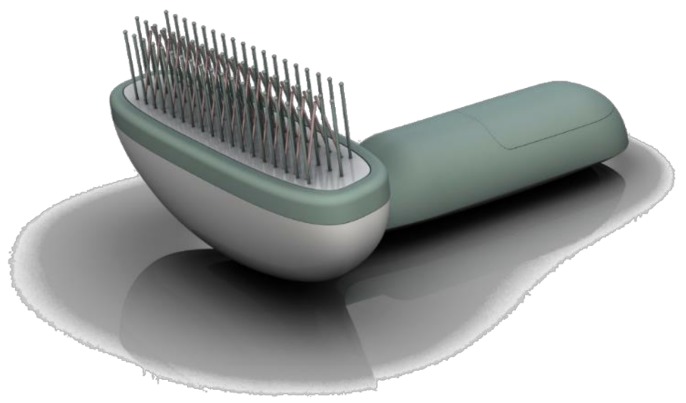
Rendered picture of the PlaCo showing three rows of spacing tines (straight tines) and two rows of active electrode tines (forming a V-shape). Design by Prof. Dipl. Ing. A. Schulz (Head of Course), Product and Industrial Design at HAWK.

**Figure 2:**
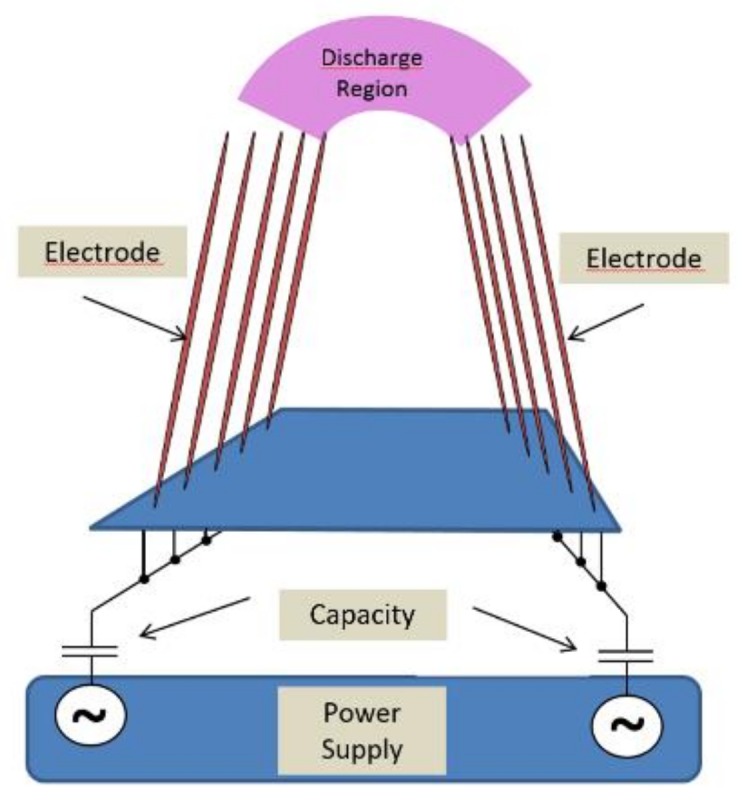
General scheme of the PlaCo setup.

A schematic of the setup used for the presented experiments is depicted in Figure 1 and Figure 2. It consists of capacitively coupled V-shaped metal electrodes used for the generation of a cold plasma at atmospheric pressure forming a comb-like device. The device is driven using a 9-V battery giving total independence from the main supplies. Using a microprocessor controlled flyback converter with a primary/secondary transformer operated in resonance, the device is operated within the parameter field as depicted in Table 1. The applied voltage is regulated varying in turn with the duty cycle. 

The presented version of the PlaCo was engineered with five rows of tines (see Figure 1). Two rows forming a V-shape are the active electrode tines. The three remaining rows consisted of nonactive tines, keeping a constant distance of the active electrodes to the skin surface. To increase the volume of the active zone, the electrode tines were interlaced relative to each other and rotated 45° perpendicular to the combing direction. This arrangement of the prongs was chosen during the design process of the device. As the midst row of spacer prongs was positioned in between the electrode prongs, they were removed to guarantee a free passing of the hair. Figure 2 is giving a general idea of the setup.

### 2.1. Test Organisms and Treatment Procedure

To investigate the effect of a plasma treatment by the comb-like device to human lice in laboratory conditions, *Pediculus humanus humanus* (lice and eggs) served as test organisms. This is a standard test organism for basic and simulated-use efficacy tests of products to control *Pediculus humanus* and thus for decades has been an approved surrogate for efficacy studies in laboratory conditions that target the head louse *Pediculus humanus capitis* [22,23]. The lice derived from the insecticide-sensitive *P. humanus humanus* strain of the German Environment Agency.

The tests presented herein were conducted using separate groups of female (treatment *n* = 65, control *n* = 60), male (treatment *n* = 30, control *n* = 60), and third instar juvenile (treatment *n* = 60, control *n* = 60) lice on human hair strands of approx. 22 cm in length with a weight of 1.5 g. The lice were sucking blood for up to 15 min on rabbits a day prior to treatment and were subsequently stored in an incubator at 32 °C and 50% relative humidity (RH). The treatments of the samples with the PlaCo device were conducted on hair strands in a horizontal position on a melamine tray using three to five lice/strand that were crawling arbitrarily throughout the hair. Using a single stroke of the device over the hair strand, the plasma was applied at a speed of approx. 2 cm/s. In the control groups, the treatment was conducted with the PlaCo without ignition. After the treatment, the test groups were incubated at a temperature of 32 °C and a relative humidity of 50% until the end of the observations. The lice were inspected 16 h after treatment. The mortality was calculated as the fraction of lice that were dead or moribund and died without recovery, related to the overall number of observed individuals. Dead lice permanently did not show any external and internal vital signs. Stimulation by light and forceps was applied. Moribund lice were lying in an irreversible back position, able to move extremities or antennae. Without external vital signs, an assessment of internal vital signs, like peristaltic of the ventriculus, was carried out.

To conduct experiments to determine the susceptibility of eggs of *P. humanus humanus* to a CAPP treatment, eggs in the early stage of embryonal development (0–2 d after deposition) and in the later stage of embryonal development eggs (4–6 d after deposition) were used. The eggs were deposited by females on a single natural hair. The hairs with single eggs were introduced directly into the plasma for approx. 1 s. The experiments were conducted at a temperature of approx. 22 °C and a relative humidity of approx. 56%. Two groups of untreated eggs served as control groups. Each of the groups within the trial consisted of 30 eggs. The mortality of the eggs was calculated as the fraction of the eggs that died (without full or incomplete hatch of larvae) related to the number of observed eggs.

The binomial 95% confidence interval of the mortality was calculated according to Clopper–Pearson. The Fisher’s exact test served to estimate the significance of the difference of the mortality (α = 0.05).

### 2.2. Spectroscopic Temperature Measurements and Calculations

In order to characterize the plasma discharge regarding reduced electrical field strength and rotational, vibrational, and electron temperature, optical emission spectroscopy (OES) was used. When measuring and calculating these characteristics, it is possible to get an idea of the collisional properties of the plasma in discussion and therefore of the occurring species (e.g., reactive oxygen/nitrogen species (RONS)). These RONS are known to be of interest when applying atmospheric pressure plasma to biological surfaces. These characteristic parameters were measured and calculated to describe the device comprehensively and to give an idea of the parameter space in which the device was operating.

The spectra were obtained using an Èchelle-Spectrometer (Aryelle-Butterfly 400; LTB Lasertechnik Berlin GmbH, Berlin, Germany) with a resolution of <80 pm. The spectrometer was calibrated to wavelength and relative intensity. The optical fiber was placed perpendicular to the filaments ignited between the active electrodes. Ten spectra were taken, each with an exposure time of 30 s. They were then dark corrected and integrated to increase the signal-to-noise ratio for further analysis. All spectra were taken at room temperature and ambient air (25 °C; 59% RH).

To determine the rotational temperature, the rotational structure of the 0–0 vibrational transition of the second positive system (C^3^Π_u_-B^3^Π_g_) of nitrogen of the measured spectra was compared to a database of simulated spectra using a routine as presented by Peters et al. [24]. The simulated data were produced using Specair 3.0 (SpectralFit, Antony, France), and all further calculations were conducted using MATLAB R2015b (The MathWorks GmbH, Ismaning, Germany). The spectra were examined in the wavelength ranges of 333.8–337.1 nm and 334.0–335.5 nm to determine a mean temperature and a maximum temperature as presented by Helmke et al. [25]. Vibrational temperatures were determined applying the Boltzmann plot [24]. To derive the electrical field strength, mean electron energy and electron temperature calculations were conducted as discussed by Peters et al. [24,26] using the method described in [27,28,29]. To achieve a high resolution, the integrated emission intensity of the complete rotational-vibrational bands (334.0–337.2 nm for the second positive system of nitrogen, respectively, 389.5–391.5 nm for the first negative system of nitrogen, respectively) was used to calculate the intensity ratios.

### 2.3. Safety Assessment

As the plasma source used was developed as a possible end-user product, the user safety of the device had to be determined. Three focal points were selected in order to assess the safety of a plasma source being applied on humans or mammals in general. The first of these was the occurring ozone concentration, which is generated prominently when working in oxygenic gases under moderate temperatures [30,31]. Secondly, the emission of UV-light had to be addressed. The third parameter which concerned operational safety was the so-called patient leakage current (PLC). Plasma sources that work based on the concept of dielectric barrier discharges (DBD) operate with high electric potentials. The amplitudes of the applied voltages can range from a few kilovolts up to some 10 kV, and when used as medical or hygiene devices, work in close proximity to the skin of patients. When working in these conditions, low leakage currents are crucial for the safe electrical applicability of the devices.

#### 2.3.1. Ozone Concentration Measurements

Plasma sources operating in ambient air at atmospheric pressure realized as a dielectric barrier discharge or similar are known to produce ozone (CAS-Nr.: 10028-15-6). Humans are able to sense very low concentrations of only a few 0.01 ppb, which lies well below the permissible levels that are set as limits for exposure by different governmental organizations. The US Occupational Safety and Health Administration (OSHA (Occupational Safety and Health Administration, USA)) determines a limit of 0.1 ppm over an 8-h time weight average (TWA) and a short-term exposure level (STEL) of 0.3 ppm. The Control of Substances Hazardous to Health Regulations (COSHH (Control of Substances Hazardous to Health Regulations, GB)), as a statutory instrument in Britain, operates under different workplace exposure limits [32]. Here, the STEL is given to be 0.2 ppm. The COSHH does not provide any information concerning the TWA. The different limits of OSHA and COSHH are presented in Table 2. To assess the ozone concentration levels which were generated during the plasma treatment, the measurements were taken using the Ozone Monitor Model 106-L, from 2B Technologies, Inc. (Boulder, CO, USA). These ozone monitors perform with a detection limit of 1.5 ppb to an upper limit of 100 ppm. Precision and accuracy are given to be higher than 1.5 ppb or 2% of reading. The ozone monitor was operated at a flow rate of approx. 1 L/min. Cell temperature (29 °C) and voltage of the photodiode (1.8 V) were constant over the measurement period as well as the cell pressure (946 Torr).

The measurements were conducted in a very large open room (>400 m^3^) at approx. 20 °C. This setup provides the opportunity to omit possible concentration effects during the measurement periods of 5 min. As the measurements were conducted in an open room, the occurrence of slight air drafts during the measurements was unavoidable. This circumstance was accepted as it reflects on the reality of the later real-life treatment scenarios.

The distance from the user to the source plays a vital role when assessing the ozone concentrations. Therefore, the measurements were performed at three different distances from the source, starting from 0 cm (interelectrode section and worst-case scenario) over 1 cm up to 10 cm (forehead–nose distance). The distance of 10 cm was estimated as the average approx. forehead/temple to nose distance when applied on the human head.

#### 2.3.2. UV Emissivity Measurements

To determine the UV radiation emitted by the used plasma source, a measuring setup was employed as described in detail by Helmke et al. [25] using an absolute calibrated detector head UV-3719-4a(z) operated on a X1-1 optometer from Gigahertz-Optik (Tuerkenfeld, Germany). The detector head had a cosine-corrected field of view taking into account that human skin acts as a Lambertian surface (i.e., an ideally scattering surface). As in the present case the plasma of the PlaCo was ignited between two electrodes, no further counter electrode (e.g., indium tin oxide (ITO) covered glass, as presented by [25]) was necessary for measurements concerning this setup. The omission of this counter electrode afforded an easier measurement setup where no absorption of any further material in the direct beam path had to be considered.

An emission spectrum of the PlaCo was recorded using an AvaSpec-ULS3648-Usb2 from Avantes BV (Apeldoorn, The Netherlands).

To assess the UV emission of the PlaCo device and to calculate a maximum exposure limit, all of following calculations were conducted following ISO/CIE 19476 [33] and the *ICNIRP Guidelines on Limits of Exposure to Ultraviolet Radiation* [34]. These calculations are necessary when applying an integral radiometer to correct the detector readings from its spectral sensitivity to a user-chosen weighting function. In our case, the correction related to the relative spectral effectiveness *S*(*λ*) of UV radiation on human skin and yielded the reciprocal spectral mismatch correction factor (SMCF) a*. To calculate the correct SMCF for the spectra of the PlaCo, four sets of information were needed: (1) an emission spectrum from the PlaCo itself; (2) the emission spectrum of the calibration lamp used by Gigahertz-Optik for the initial calibration; (3) the relative spectral effectiveness of the human skin calculated according to [34]; and (4) the relative spectral sensitivity of the UV-3719-4a(Z) detector head. The second and fourth datasets were kindly provided by Gigahertz-Optik. With this data, the effective irradiance *E_eff_* (W m^−2^) of the PlaCo was calculated as follows:

(1) $$E_{eff}=\frac{E_{meas}}{a^{*}}$$

with

(2) $$a^{*}=\frac{\sum E_{\mathrm{cal},\lambda }\cdot S\left(\lambda \right)\cdot \Delta \lambda }{\sum E_{\mathrm{cal},\lambda }\cdot Z\left(\lambda \right)\cdot \Delta \lambda }\cdot \frac{\sum E_{\lambda }\cdot Z\left(\lambda \right)\cdot \Delta \lambda }{\sum E_{\lambda }\cdot S\left(\lambda \right)\cdot \Delta \lambda }.$$

Here, *E_meas_* is the measured irradiance (W m^−2^) of the detector, *E_cal_*_,*λ*_ is the spectral irradiance of the calibration lamp at wavelength *λ*, *S*(*λ*) is the relative spectral effectiveness (unitless) and is calculated according to [34] in the range of 250–400 nm, *E_λ_* is the spectral irradiance of the PlaCo at wavelength *λ* (W m^−2^ nm^−1^), and *Z*(*λ*) is the relative spectral sensitivity of the detector head (unitless). The proper determination of the effective irradiance (*E_eff_*) of a broadband source is crucial when the goal is safely minimizing the long-term risks during skin exposure to a UV radiation source.

In [34], the (effective spectrally weighted) limit value for UV exposure to the human skin is stated as 30 J m^−2^/day. This allows the calculation of the maximum duration of exposure per day *t_max_*:

(3) $$t_{max}=\frac{30\;{J\;m}^{-2}}{E_{eff}}.$$

#### 2.3.3. Patient Leakage Current Measurements

The assessment of the PLC of the PlaCo was performed following DIN EN 60601-1. Therein, it is stated that the patient leakage current must not exceed 10 mA at any time. The limits for PLC are given to be max. 10 µA at normal condition (NC) and max. 50 µA at single fault condition (SFC). These limits consider the PLC setup, using a 9-V battery as the power source, to function as a body float setup (BF) following the same DIN.

**Figure 3:**
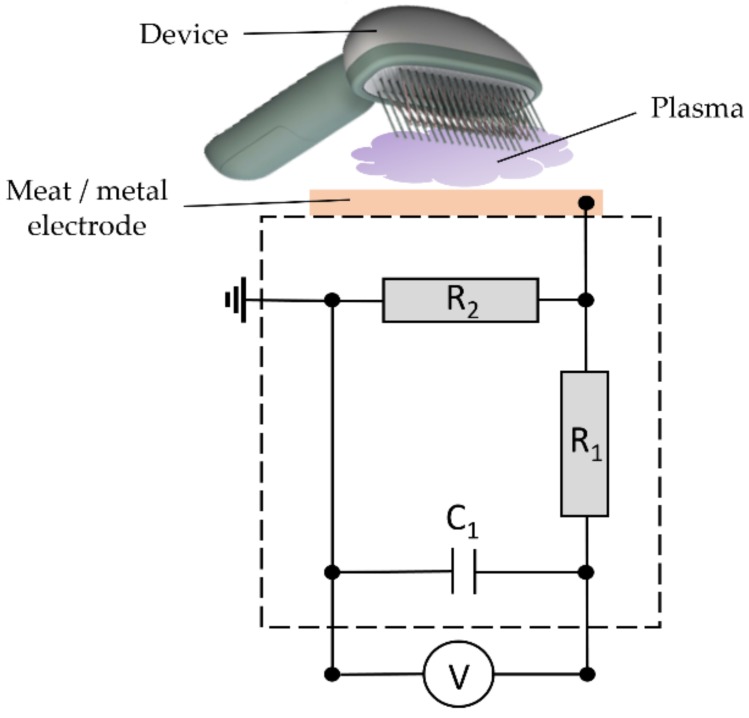
Schematic diagram for patient leakage current (PLC) measurements following DIN 60601-1; R_1_ = 10 kΩ; R_2_ = 1 kΩ; C_1_ = 10 nF; V measured by ELV DT-8000 voltmeter.

The considered case for this measurement was the measuring section of patient contact point to ground. The measurement arrangement (patient model) is shown in Figure 3. For the presented measurements, the DT-8000 voltmeter (ELV AG, Leer, Germany) was used, following the DIN. The occurring current equivalent voltage drop over C_1_ was measured, which can be considered equivalent to the voltage drop over R_2_, due to the high resistant ratio between R_1_ and R_2_. Functioning as scalp models, two different measurement setups were used—the first using a piece of pork meat, the second with an aluminum electrode, respectively. Introducing the meat into the circuit gave a more natural ignition behavior of the PlaCo compared to the metal reference electrode, which resulted in much hotter, spark-like discharges. Four different cases could be measured for every electrode. The first case may be considered the “standard” case, where the electrode prongs were kept at a constant distance of 2 mm using the spacer prongs. The second case, called “worst case” here, was simulated with one side of the electrodes touching the test electrode completely. Here, no plasma was ignited but a current did flow. These two cases were subdivided into two further cases. In case three, the PLC of the device was measured using only the battery (floating potential), resembling the real-life operating mode. In case four, the negative side of the power source was grounded to bring it to a defined potential level.

## 3. Results

### 3.1. Plasma Comb Efficacy Tests on Lice

Depending on the duration and mode of contact to the plasma, the immediate effects of the treatment to the lice varied from no visible effects over an abnormal movement to visible internal damage within 20 min to 6 h post-treatment. When heavily impaired within the first minutes after treatment, a perspiration-like release of liquid apparently through the cuticle was observed. This plasma-induced impairment was reproducible and was often followed by a leakage of the digested blood from the ventriculus into the surrounding hemolymph. First observation measurements using an imaging technique called optical coherence tomography (OCT) revealed a clear rupture incident detectable only a few minutes after plasma treatment. This rupture seems to occur in the joint between the mid-gut and head section, leading to internal damage of the intestines, and often occurs within the first few minutes up to 6 h subsequent to plasma treatment (OCT results not presented). Dead lice often showed desiccated and partially deformed features and a progressive invasion of blood residues from the ventriculus area into the thoracal hemolymph region and into the extremities. It is apparent that the necessary time period, elapsing to reach exitus, was determined by the quality of the treatment (full exposure/partial exposure).

**Figure 4:**
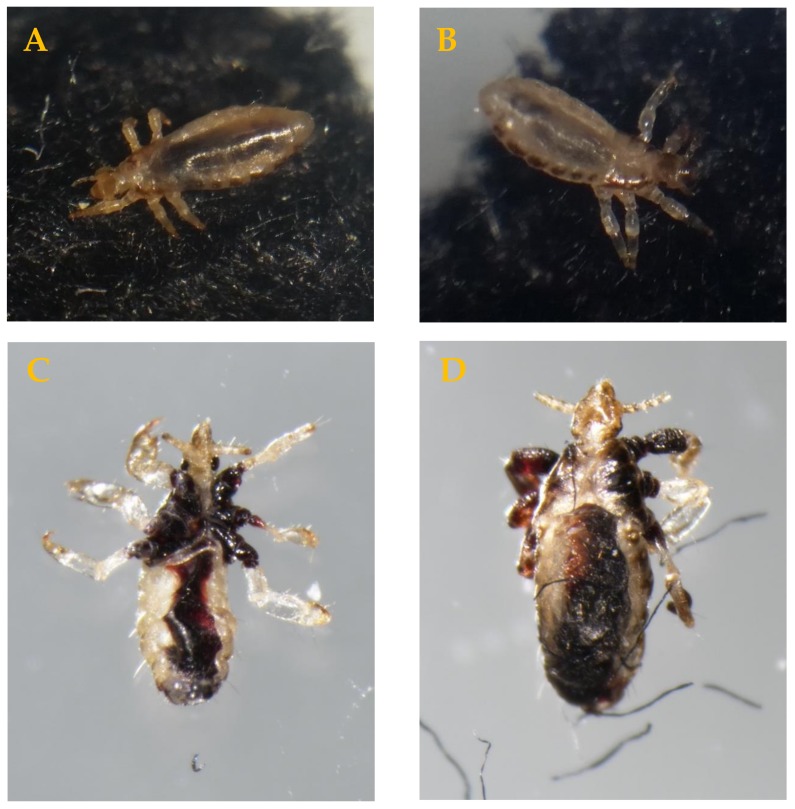
(**A**) Third instar of *Pediculus humanus humanus* directly after plasma treatment. The movement was impaired. (**B**) Third instar of *P. humanus humanus* directly after plasma treatment. Perspiration occurs with impairment of movements. (**C** and **D**) Damage pattern in third instar lice 6 h subsequent to plasma treatment. Depending on quality of treatment, this damage pattern began to occur a few minutes after treatment, showing transgression of the human blood from digestive tract to the surrounding hemolymph. After 6 h, clearly visible exsiccation and deformation of head, body, and extremities occurred. The overall lengths of the depicted lice are approx. 2.3 mm.

The damage pattern occurring in the treated lice is depicted in Figure 4.

**Figure 5:**
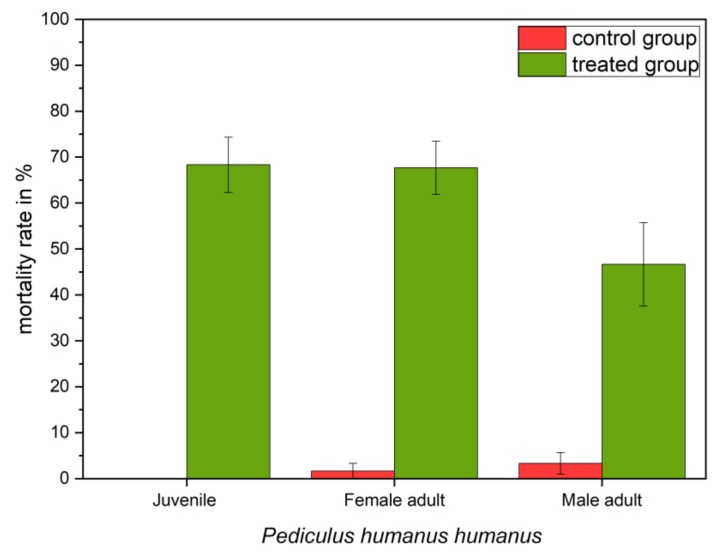
Mortality after one single transition of the PlaCo device through a 22-cm hairpiece, comparing the effect of developmental stage and sex on the effectivity of the treatment. The damage of the individuals was assessed 16 h after their last feeding.

As depicted in Figure 5, when simulating a treatment procedure at 2 cm/s (see Section 2.1), the mortality of the third instar larvae in the treatment group amounted to approx. 68.3% (50.0; 79.7) (95% CI). The group of adult females showed a mortality rate of approx. 67.7% (54.9; 78.8) (95% CI), which was comparable with the mortality rate of juvenile group. When looking at the adult group of treated male individuals, the mortality rate was lower, revealing a mortality of approx. 46.7% (28.3; 65.7) (95% CI). Only a small percentage of dead lice were observed in the control groups (juveniles: 0%; female imagines: 2%, male imagines: 3%).

**Figure 6:**
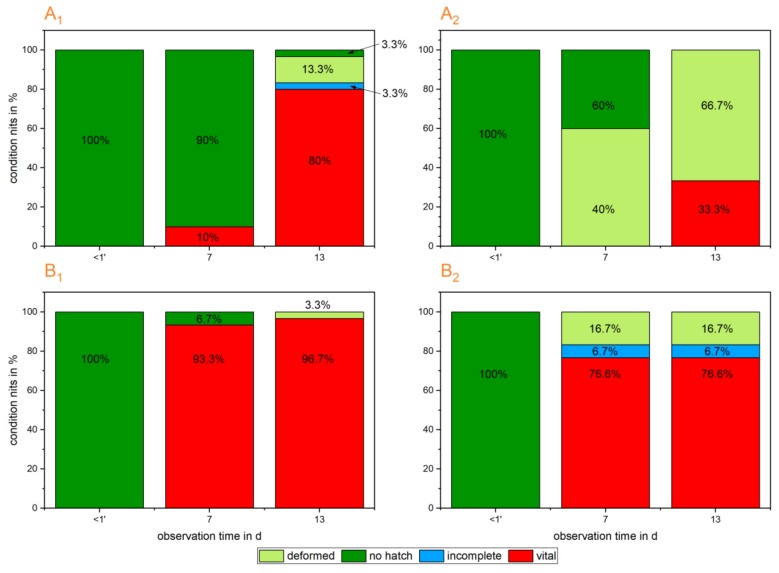
Depicted are the hatch rates of 0–2 d old eggs (A_1_: ref. group; A_2_: treated group) and the hatch rates of 4–6 d old eggs (B_1_: ref. group; B_2_: treated group). The assessment of the egg development considers different states. These are: **no hatch**: intact egg, no hatch occurred; **deformed**: deformed egg, no hatch occurred; **incomplete**: partially hatch occurred, juveniles opened the operculum and started moving out of the egg but did not finish the procedure; **vital**: embryonal development and hatch complete.

The treated eggs demonstrated varying mortalities after the plasma treatment. Younger eggs (0–2 d) showed a higher mortality of 66.7% (42.7; 82.7) than the older (4–6 d) eggs, with 16.7% (5.6; 34.7) (CI) at the end of the observation period (*p* < 0.001). The control group of younger eggs (0–2 d) revealed a mortality of 13.3% (5.6; 34.7) and the control group of the older eggs (4–6 d) a mortality of 3.3% (0.1; 17.2). For young eggs (0–2 d), the effect of the treatment was significant compared to the control group (Fisher’s exact test; eggs 0–2 d: *p* < 0.001; eggs 4–6 d: n.s.). The state of incomplete hatch was not included to the mortality of eggs, as the hatching process and the fitness of the first instar can be influenced by other factors, not only the treatment. Figure 6 shows a comparison of the differences in the hatch rates of younger and older plasma-treated eggs compared to their respective reference groups.

### 3.2. Temperature Measurement

The calculation of the temperatures resulting from the comparison of simulated with measured spectra, as presented in Section 3.1, resulted in temperatures as presented in the following (Table 3).

### 3.3. Safety Assessment

To pose not only a user-friendly but also healthy and nontoxic treatment method and to ensure user safety, plasma devices for medical applications have to be assessed to determine occurring concentrations of ozone and the emitted UV light as well as for occurring patient leakage currents. In the following subsections, measurement results and calculations of these potential health risks are presented.

#### 3.3.1. Ozone Concentration Measurements

**Figure 7:**
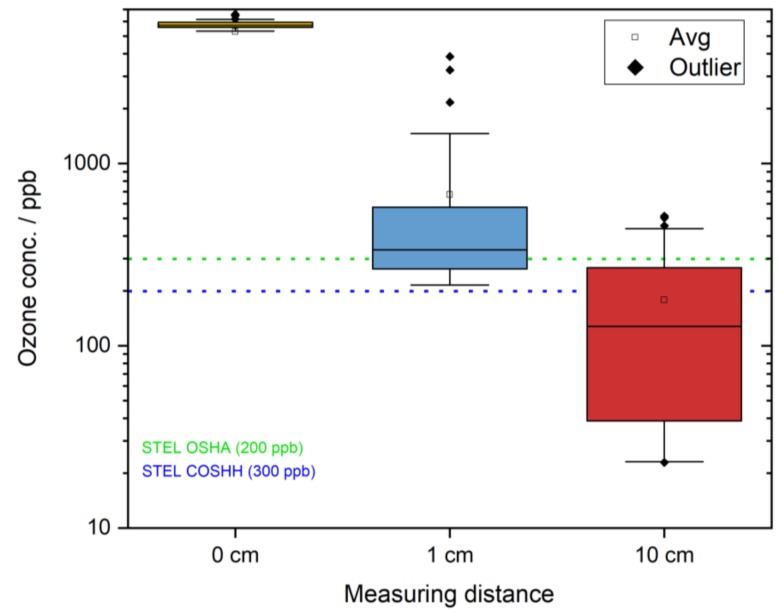
Box plot of ozone concentrations measured at three different distances of the plasma source (logarithmic scaling).

As depicted in Table 4, the ozone limits as introduced by OSHA and COSHH were met when the plasma comb was operated on a human head measured in a distance of 10 cm (average distance from hairline to tip of the nose). The ozone concentrations measured at this distance satisfied the limits of OSHA by a factor of 0.59, and the COSHH limits were met by factor 0.89. Figure 7 displays the decreasing ozone concentrations with regard to the sampling distance.

#### 3.3.2. UV Emissivity of Plasma Comb

The calculation of the reciprocal SMCF depends on the wavelength range considered. The wavelength range was limited from 250 to 400 nm. Below 250 nm, only the relative spectral effectiveness *S*(*λ*) was significantly different from zero, which is why none of the calculations yielded significant contributions to the summations in (2). Above 400 nm, both weighting functions, *S*(*λ*) and *Z*(*λ*), were almost zero, leading to possible neglection to summations above this threshold.

**Figure 8:**
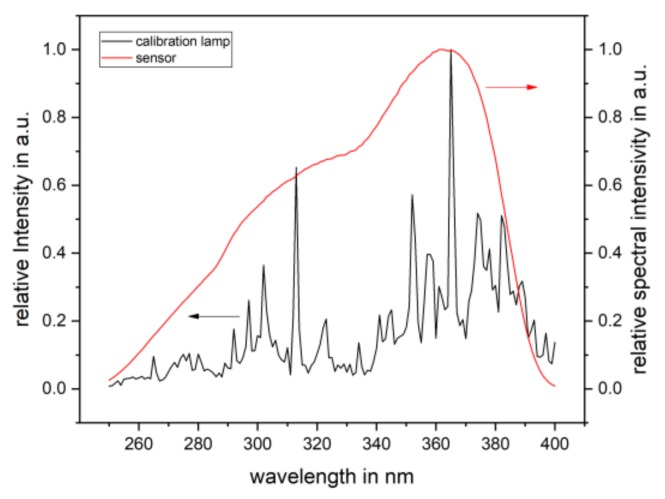
*E_cal_*_,*λ*_, relative irradiance of the calibration lamp (**black**), and *Z*(*λ*), relative spectral sensitivity of the UV-3719-4a(Z) detector head (**red**).

**Figure 9:**
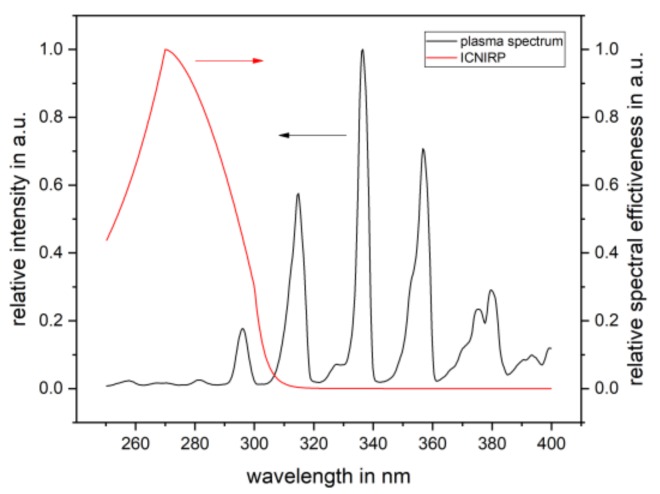
E_λ_, relative irradiance of the PlaCo device (**black**), and *S*(*λ*), relative spectral effectiveness calculated according to [21] (**red**).

The measurement with the UV-3719-4a(Z) detector revealed an uncorrected irradiance of *E_meas_* = 1.57 mW m^−2^. The calculation of the SMCF was necessary to correct this value such that it reflected the effective intensity to which human skin is exposed. The calculation presented in Section 2.3.2 used the spectral data given in Figure 8 and Figure 9. The reciprocal SMCF for the PlaCo device could be calculated to a value of a* = 1.77. The correction led to an effective irradiance of *E_eff_* = 0.89 mW m^−2^, which translated to a maximum exposure duration of *t_max_* = 9.3 h.

#### 3.3.3. Patient Leakage Current Assessment

**Figure 10:**
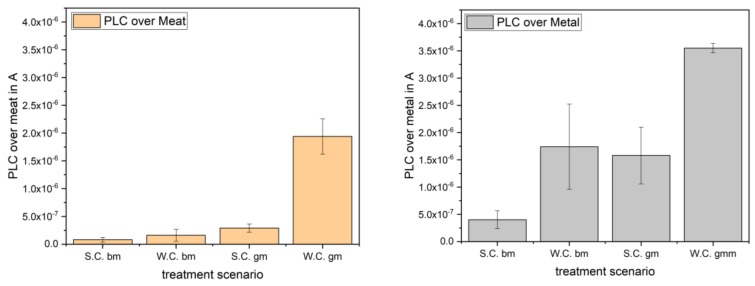
Two different materials were measured resembling the surface of the scalp. The left diagram gives the results for the meat electrode, and the right side depicts the results for the metal surface. S.C. is the standard case; no electrode prongs touched the “scalp”. W.C. stands for worst case; here, all prongs of one half of the device were touching the “scalp” (no plasma was igniting). The abbreviations for bm and gm stand for battery mode and grounded mode, respectively (compare Section 2.3.3).

The measurement of the patient leakage current performed on two different materials (mammal flesh and aluminum) and following DIN EN 60601-1 revealed patient leakage currents as depicted in Figure 10.

Following the norm for PLC measurement, the currents during the normal case must not be higher than 100 µA. The measured currents fell below this limit by a factor of more than 25×. The occurring currents lay well below the perception threshold of approx. 30 mA (at approx. 240 kHz), as studied by Chatterjee et al. [35].

## 4. Discussion

As the spectroscopic measurements revealed a rotational temperature of the filaments of the plasma of approx. 550 to 700 K, these temperatures seem quite hot at first, especially when considering T_rot_ ≈ T_gas_. It should be noted that these temperatures rise over a very short period of time, as the filaments show an estimated lifetime of a few 100 ns [36,37] when excited using 16 kVpp at 245 kHz in a 2 mm gap. When regarding the lifetime of the 0–0 vibrational transition of the second positive system (C^3^Π_u_-B^3^Π_g_) of nitrogen, determining the rotational temperature this time even reduces to 37 ns [28]. Considering these short filament lifetimes, although assuming T_rot_ ≅ T_gas_ following [38], an effective heating of tissue and hair material is considered negligible. Furthermore, the filament temperatures, that is, the small heated gas volumes, decline to room temperature within some milliseconds by heat energy transfer to the surrounding neutral gas. This reflects in the almost imperceptible application of the PlaCo on human skin. All measured and subsequently calculated temperatures are displayed in Table 3.

The damage pattern of affected lice and the mortality of juvenile and adult lice and their eggs after a single stroke on human hair with the speed of 2 cm/s or 1 s, respectively, clearly show the efficacy a CAPP produced using the PlaCo device against *P. humanus humanus* in the given conditions. The intensive water loss of the mobile lice when subjected to the plasma treatment also occurred in other insects after plasma treatment [39]. The observed deformations of the lice after a plasma treatment are not known from other chemical or physical treatment procedures to control head lice or other insects of significance to health. A transferability of the results is expected as *P. humanus humanus* is known as an approved surrogate for efficacy studies under laboratory conditions, as mentioned in Section 2.1.

The efficacy of the device when applied for a single transition was determined to be up to approx. 68%. After the treatment of the eggs, approx. 66% of the younger eggs (0–2 d) could be killed within 1 s of treatment time, whereas the older eggs were only affected on a very low level. The deactivation rates for the eggs differ strongly depending on the age of the eggs. A similar effect is known from standard chemical pediculicides, where older eggs also demonstrate a higher resilience. Considering that this was a forced test, a simulated-use test on hair strands with a higher number of eggs is necessary to estimate the efficacy against eggs in more practical conditions. The eggs have only a small size of about 0.8 mm in length and a smaller width, so the position of the egg according to the plasma might influence the effect to the egg. A small size effect could also be possible in first instar juvenile lice with a size of about 0.8–1.0 mm. These smallest stages also should be included in the simulated-use tests. Considering the given results of the eggs, at minimum, one repetitive complete treatment will be necessary at this point after 8–10 days when applying the PlaCo in a real-life situation.

We want to point out again that the presented results were obtained in conditions of a horizontal test on a flat surface using a relatively small amount of hair. An inactivation rate of 100% was not achieved using the presented test conditions. When, for example, integrated as a combing procedure during a “standard” morning routine of showering, washing, styling hair, etc., the combing process is characterized by a significantly higher amount of combing strokes (>>1). When hypothetically estimating a constant killing rate per stroke of approx. 70%, a killing rate of 99.9% could be achieved after six strokes per strand. When assuming a combing procedure of 10 min repeated in sets of three over a complete day, the necessary killing rate of 100% seems achievable.

A systematic clinical investigation taking into account a larger volume of hair, the natural contour of a human head, and a large number of combing strokes is saved for future work.

The currently presented safety assessment focused on the three main components that arose from the occurring ozone, UV radiation, and currents that flowed over the treated person to ground potential. The ozone concentration showed very high concentrations, especially when measured at its point of origination (see Figure 7). When increasing the distance from the sampling point to the source successively, the ozone concentration decreased. When measured in a relevant distance (i.e., the forehead/temple-to-nose distance), the OSHA and COSHH limits were met, leading to a possible treatment time of more than 15 min. Nonetheless, ozone concentrations subsequent to treatment can be detected by the user and can lead to an unpleasant odor perception of the treatment. A reduction of these emerging ozone concentrations should therefore be considered during future developmental adjustments of the device.

Further gases, like various forms of nitrogen oxides (NO_x_), might occur during a plasma treatment but were not under investigation within this study. As the processes of ozone and nitrogen oxide generation are contending processes [37,40], NO_x_ gases are not expected to exceed the amount of occurring ozone. When aiming at a possible market launch of plasma devices for use on humans, all occurring reactive gases should be validated.

The occurring UV intensities were measured and showed an effective irradiance of *E_eff_* = 0.89 mW m^−2^. This irradiance determined a maximum possible treatment time of 9.3 h, which exceeded the limiting factor of the ozone concentration by far.

The occurring PLCs emitted during a standard treatment were negligible, as depicted in Figure 10, even when considering the worst-case scenarios using a grounded reference potential.

## 5. Conclusions

Considering the results concerning efficacy and safety of the presented plasma device, the high potential of this source can well be demonstrated. Here, studies were conducted concerning the effect of the PlaCo device on the hair material investigating possible negative effects. The occurring UV radiation, ozone concentration, and occurring patient leakage currents fell below the thresholds given by different organizations and norms. When considering the 10-cm thresholds for ozone concentrations to be the “worst case”, the maximum possible treatment times using the presented PlaCo device might even be greater than the calculated 15 min. When aiming at bringing the device into the market the ozone concentrations should be assessed again as well as possibly occurring NO_x_ concentrations, considering the variable experimental conditions. Based on the presented results, treatment times of more than 10 min for a complete adult head seem unnecessary. Further enhancements of the PlaCo device should consider a reduction of the occurring ozone concentrations and a further enhancement of the electrode configuration. Here, a spread of the electrode rows in different distances from the scalp, giving a better performance within three dimensions, might enhance its performance further.

The presented method may be developed into an effective and promising alternative approach to chemical treatment agents. The possibility for total omission of pediculicides with pharmacological mode of action might even reveal positive effects concerning skin health, as the chemical agents in use tend to induce post-treatment skin irritation for a recognizable period of time. The symptoms occurring after a severe infestation of head lice such as dandruff, eczema, etc., might even be mitigated by the plasma itself.

## Figures and Tables

**Table 1:** Parameter settings of the plasma comb (PlaCo) device.

Input Parameter	Value
Electrical power	≈590 mW
Discharge gap	≈4 mm
Appl. voltage	≈16 kV (p-p)
Natural frequency	≈245 kHz
Puls rep. rate	≈0.6–1 kHz
Waveform	decaying sine

**Table 2:** Ozone concentration maxima following regulatory bodies of Great Britain and the United States.

	O_3_-max. OSHA in ppm	O_3_-max. COSHH in ppm
STEL (15 min)	0.3	0.2
TWA (8 h)	0.1	/

**Table 3:** Rotational, vibrational, and electron temperature and reduced electric field strength as determined by optical emission spectroscopy (OES).

	Values
Mean rotational temperature, *T*_rot, mean_	595 ± 50 K
Max. rotational temperature, *T*_rot, max_	660 ± 50 K
Vibrational Temperature, *T*_vib_	4110 ± 612 K
Reduced Electric Field, *E*_R_	209 ± 12 Td
Mean Electron Energy, *ε*	5.5 ± 0.2 eV
Electron Temperature, *T*_e_	42.7 × 10^3^ ± 1.8 × 10^3^ K

**Table 4:** Ozone concentration during application of the plasma comb measured in three different distances.

Nozzle Distance	Median O_3_ Conc./ppb	OSHA Multiple	COSHH Multiple
0 cm	5729.35	≈17.6×	≈26.4×
1 cm	335.35	≈2.25×	≈3.4×
10 cm	127.7	≈0.59×	≈0.89×
