# Erratum: ten Bosch et al. Cold Atmospheric Pressure Plasma Comb—A Physical Approach for Pediculosis Treatment

**DOI:** 10.3390/ijerph17020450

**Published:** 2020-01-09

**Authors:** Lars ten Bosch, Birgit Habedank, Dominik Siebert, Julia Mrotzek, Wolfgang Viöl

**Affiliations:** 1 Faculty N, University of Applied Sciences and Arts HAWK, Von-Ossietzky-Strasse 99/100, 37085 Göttingen, Germanyjulia.mrotzek@hawk.de (J.M.); wolfgang.vioel@hawk.de (W.V.); 2 German Environment Agency, Corrensplatz 1, 14195 Berlin, Germany

Due to an error during production and a corrupted data set, Section 3.3.1 in the result section of the published paper [1] was displaying incorrect data. A corrected version of the section is provided below.

Importantly, these changes do not modify the significance and the related conclusions in any way. The authors would like to apologize for any inconvenience to the readers caused by this error.

## 3.3.1. Ozone Concentration Measurements

As depicted in Table 4, the ozone limits as introduced by OSHA and COSHH were met when the plasma comb was operated on a human head measured in a distance of 10 cm (average distance from hairline to tip of the nose). The ozone concentrations measured at this distance satisfied the limits of OSHA by a factor of 0.01, and the COSHH limits were met by factor 0.015. Figure 7 displays the decreasing ozone concentrations with regard to the sampling distance.

## Figures and Tables

**Figure 7:**
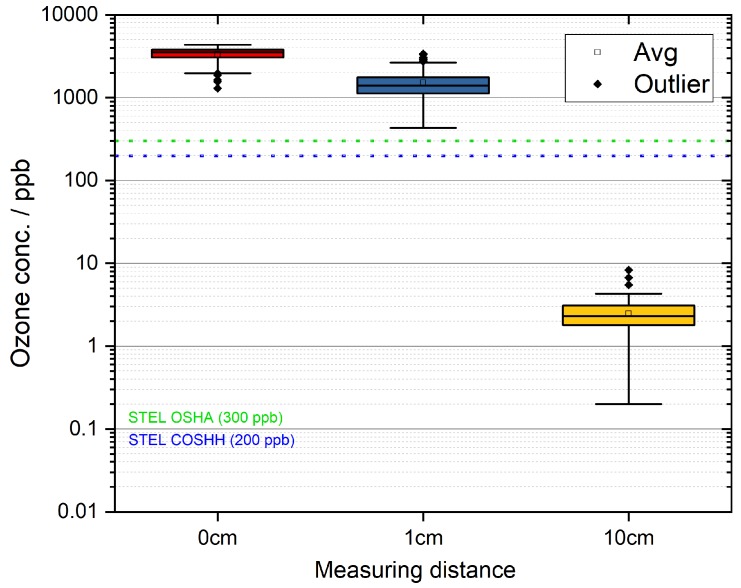
Box plot of ozone concentrations measured at three different distances of the plasma source (logarithmic scaling).

**Table 4:** Ozone concentration during application of the plasma comb measured in three different distances.

Nozzle Distance	Median O_3_ Conc./ppb	OSHA Multiple	COSHH Multiple
0 cm	3348	≈11.2×	≈16.7×
1 cm	1562	≈5.2×	≈7.8×
10 cm	2.6	≈0.01×	≈0.015×
